# Comparison of Electrothermal Ablation and Electrolyte Plasmalization Devices Based on the Mechanical Properties of Anterior Cruciate Ligament Femoral Attachment Following Partial Debridement: A Biomechanical Study Using a Porcine Model

**DOI:** 10.7759/cureus.47911

**Published:** 2023-10-29

**Authors:** Takahiro Nishimura, Tsuneari Takahashi, Katsushi Takeshita

**Affiliations:** 1 Department of Orthopaedics, Jichi Medical University, Shimotsuke, JPN; 2 Department of Orthopaedic Surgery, Ishibashi General Hospital, Shimotsuke, JPN

**Keywords:** porcine model, anterior cruciate ligament (acl), biomechanical study, lectrolyte plasmalization, electrothermal ablation

## Abstract

Purpose

This study aimed to clarify whether differences in ablation devices used in the knee joint during partial debridement of the proximal end of the femoral attachment of the anterior cruciate ligament (ACL) affect the mechanical properties of the femur-ACL-tibia complex.

Methods

Electrothermal ablation was generated from Vulcan for the left knees, and radiofrequency ablation was generated from Werewolf Flow 50 Wand for the right knees. The probes were set to the default setting of 120 W and 150 W for Vulcan and Werewolf Flow 50 Wand, respectively. To mimic partial debridement in remnant tissue-preserving (RTP) ACL reconstruction, the bipolar ablation mode and serpentine movements were employed while in contact with the femoral fan-like extension fibers of the ACL. To simulate the arthroscopic environment, the model was immersed in a saline solution. The probes were applied for 60 s, and their biomechanical properties were evaluated.

Results

A significant difference was observed in the upper yield load between the two groups (Vulcan group, 107.1 ± 93.4 N; Werewolf group, 177.9 ± 108.8 N; *P *= 0.045). However, no significant differences were noted in linear stiffness (Vulcan group, 47.6 ± 30.9 N/mm; Werewolf group, 50.1 ± 30.5 N/mm; *P *= 0.85), maximum load (Vulcan group, 276.2 ± 171.8 N; Werewolf group, 397.7 ± 150.8 N; *P *= 0.26), or elongation at failure (Vulcan group, 6.1 ± 0.9 mm; Werewolf group, 11.6 ± 10.4 mm; *P *= 0.20) between the two groups.

Conclusion

The mechanical properties of the ACL after partial ACL femoral attachment debridement for RTP-ACL reconstruction were better when an electrolyte plasmalization device was used. When performing RTP-ACL reconstruction, surgeons must consider that the device used for partial femoral ACL stump debridement may affect the mechanical properties of the ACL remnant tissue.

Clinical relevance

When performing RTP-ACL reconstruction, surgeons must consider that the device used for partial femoral ACL stump debridement may affect the mechanical properties of the ACL remnant tissue.

## Introduction

Arthroscopic surgery of the knee joint is widely performed during anterior cruciate ligament (ACL) reconstruction, and before the creation of bone tunnels, tissue ablation is performed to obtain better visibility. Femoral tunnels are created following partial debridement of the proximal end of the remaining femoral stump using a shaver, and electrothermal ablation is performed using electrolyte plasmalization devices, particularly for remnant tissue-preserving (RTP) ACL reconstruction [1,2].

Radiofrequency (RF) devices are based on the principle of destroying soft tissues through electrolyte plasmalization, which increases the temperature of the perfusate [3]; however, a study reported the risk of chondrocyte damage [4]. Within the scope of our literature search, no studies have compared the effects of electrothermal ablation and electrolyte plasmalization devices on the degradation of the mechanical properties of the knee intra-articular ligaments. The mechanical properties of the ACL following partial ACL femoral attachment debridement for RTP-ACL reconstruction might affect postoperative clinical outcomes, particularly anterior stability. Thus, we hypothesized that differences in the ablation devices used in the knee joint during partial debridement of the proximal end of the ACL femoral attachment affect the mechanical properties of the femur-ACL-tibia (FAT) complex. In this study, we aimed to clarify the abovementioned hypothesis biomechanically using a porcine knee.

## Materials and methods

**Figure 1:**
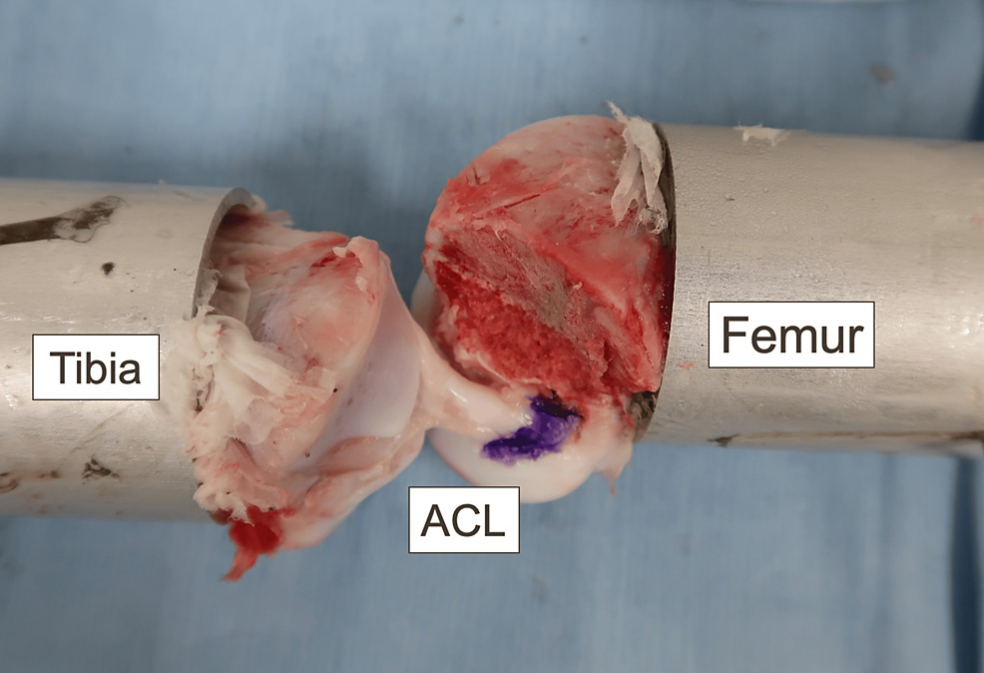
The femur–ACL–tibia complex of the right knee potted in aluminum tubes Purple painted area indicates fan-like extension fibers of the ACL. ACL, anterior cruciate ligament

This study used seven pairs of fresh porcine knees (aged six months, weighing 38-52 kg, San-S breeding [Funabashi, Japan]). The entire knee joint was harvested with the joint capsule intact. All connective ligaments and capsules around the knee joint were removed, except for the native ACL. For better visualization of the femoral ACL attachment, the distal medial condyle of the femur was also dissected. All paired right and left FAT complexes were potted in aluminum tubes [5-10] (Figure 1).

**Figure 2:**
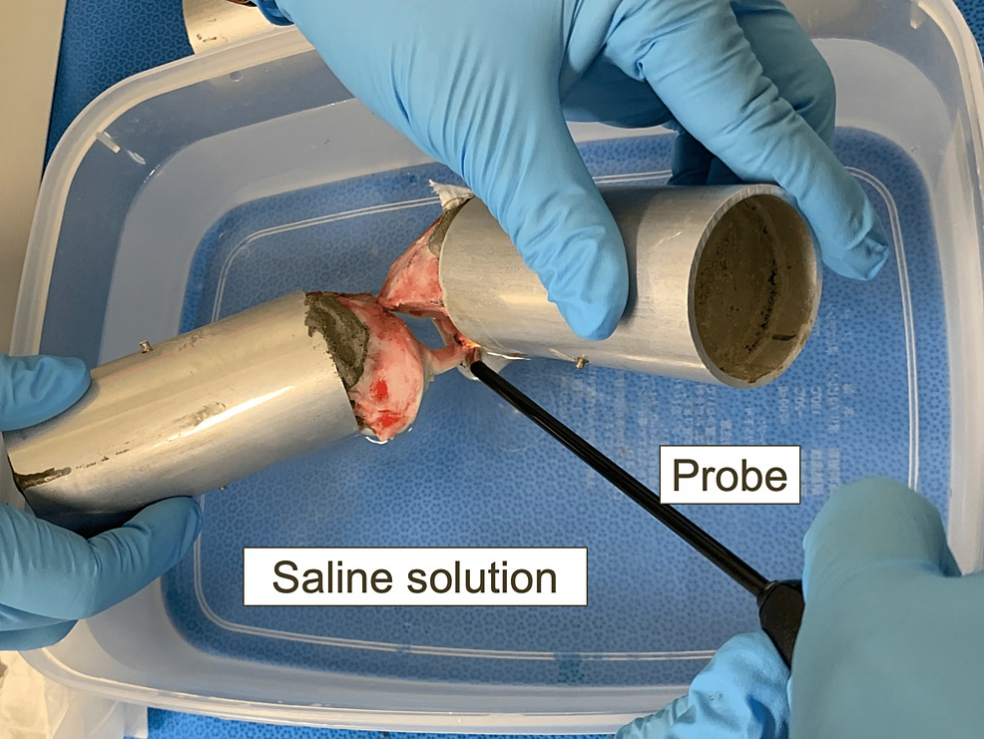
Partial debridement of the anterior cruciate ligament femoral attachment Partial debridement of the anterior cruciate ligament femoral attachment was performed in a saline solution to simulate the arthroscopic environment. The probe was applied to fan-like extension fibers perpendicularly for 60 s under default settings.

Electrothermal ablation was generated from Vulcan (Smith and Nephew, Inc., Andover, MA, USA) for the left knees, and RF ablation was generated from Werewolf Flow 50 Wand (Smith and Nephew, Inc.) for the right knees [11,12]. The probes were set to the default setting of 120 W and 150 W for Vulcan and Werewolf Flow 50 Wand, respectively. To mimic partial debridement in RTP-ACL reconstruction in patients with Crain 3 ACL remnant, the bipolar ablation mode and serpentine movements were employed while in contact with the femoral fan-like extension fibers of the ACL [1,13]. The border between the ACL mid-substance [14] and the fan-like extension fibers was determined according to the ACL fold during knee flexion [13]. No pressure was applied, and no fluid flow was used during partial debridement. We ensured that the probe perpendicular to the fan-like extension fibers did not damage the mid-substance of the ACL throughout the procedure. To simulate the arthroscopic environment, the model was immersed in a saline solution [15]. The probe was applied for 60 s [16], and biomechanical changes were evaluated immediately (Figure 2).

Structural properties of the FAT complex

**Figure 3:**
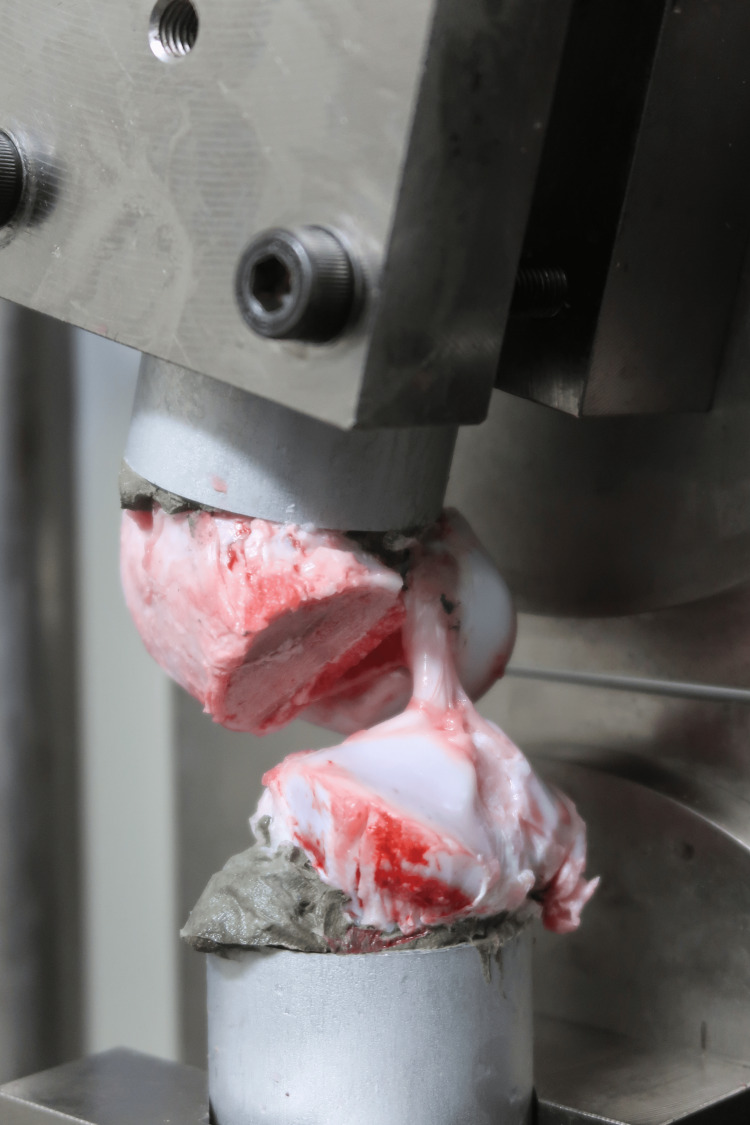
The femur–anterior cruciate ligament–tibia complex was mounted on a tensile tester using a set of specially designed grips A tensile load was applied to the anterior cruciate ligament in parallel with the long axis.

The FAT complex specimens were mounted on a tensile tester (Tensilon RTG-1250, Orientec Co., Ltd., Tokyo, Japan) using a set of specially designed grips to ensure that a tensile load was applied to the ACL in parallel with the long axis (Figure 3).

This measurement system was identical to that used in previous biomechanical studies on large animals [5-10, 17]. The tibia was fully extended. Before the test, the specimen was preconditioned with a static preload of 5 N for 10 minutes, followed by 10 cycles of loading and unloading (3% strain) with a cross-head speed of 20 mm/minutes. Subsequently, each specimen was stretched to failure under the same conditions, with preconditioning at a cross-head speed of 50 mm/minutes, and a tensile load was applied parallel to the long axis of the ACL. Load-extension curves were constructed using Tensilon Advanced Controller for Testing (Orientec Co., Ltd.). The structural properties of the FAT composites, including upper yield load, maximum load, linear stiffness, and elongation at failure, were calculated using a software. The upper yield load was defined as the point at which plastic deformation occurs, as previously reported [18,19]. Failure modes in tensile testing were also observed and recorded.

Statistical analysis

A priori power analysis was performed using G* Power 3.1 (Franz Paul, Kiel, Germany) [20]. To test the hypothesis, the sample size was calculated with a power of 80% and an effect size of 1.3. All data are presented as mean ± standard deviation. Fisher’s exact test was performed to evaluate the difference in failure modes between the two groups. All statistical analyses were performed using EZR [21]. P-values of <0.05 were considered to indicate statistical significance.

## Results

Changes in length during cyclic loading

Changes in length during cyclic testing were 1.9 ± 1.2 and 1.5 ± 0.9 mm in Vulcan and Werewolf groups, respectively. No significant difference was observed between the two groups (P = 0.23) (Table 1).

**Table 1:** The structural properties of the femur-anterior cruciate ligament-tibia complexes. Data are expressed as the mean (standard deviation).

Parameters	Vulcan (n=7)	Werewolf (n=7)	P-value
Length change	1.53 (0.94)	1.25 (0.64)	0.41
Upper yield load (N)	118.5 (96.9)	199.7 (101.0)	0.046
Linear stiffness (N/mm)	53.3 (29.6)	56.6 (27.5)	0.83
Maximum load (N)	308.1 (163.8)	350.1 (90.5)	0.57
Elongation at failure (mm)	6.23 (0.87)	11.8 (11.4)	0.27

Biomechanical evaluations of the FAT complex

A significant difference was observed in the upper yield load between the two groups (Vulcan group, 107.1 ± 93.4 N; Werewolf group, 177.9 ± 108.8 N; P = 0.045). Conversely, no significant differences were noted in linear stiffness (Vulcan group, 47.6 ± 30.9 N/mm; Werewolf group, 50.1 ± 30.5 N/mm; P = 0.85), maximum load (Vulcan group, 276.2 ± 171.8 N; Werewolf group, 397.7 ± 150.8 N; P = 0.26), or elongation at failure (Vulcan group, 6.1 ± 0.9 mm; Werewolf group, 11.6 ± 10.4 mm; P = 0.20) (Table 1) between the two groups.

Failure mode in tensile testing

**Figure 4:**
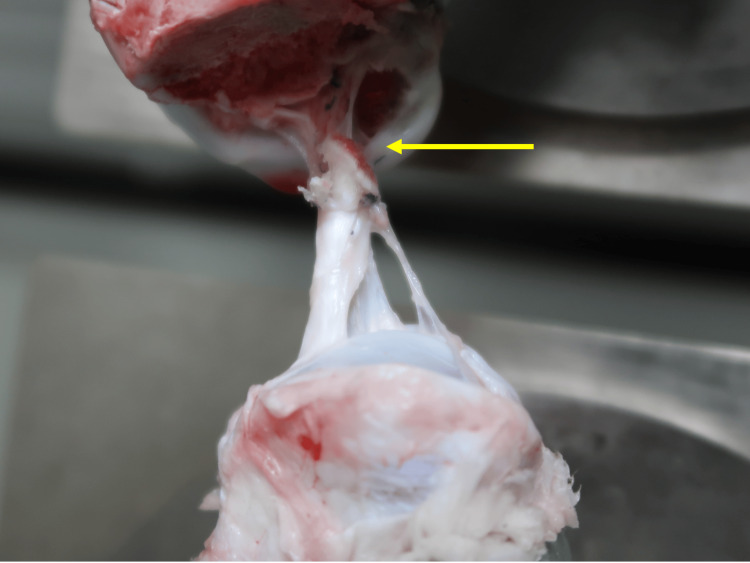
Avulsion at the femoral side of the anterior cruciate ligament attachment after tensile testing Yellow arrow indicates avulsion at the femoral side of the anterior cruciate ligament attachment.

**Figure 5:**
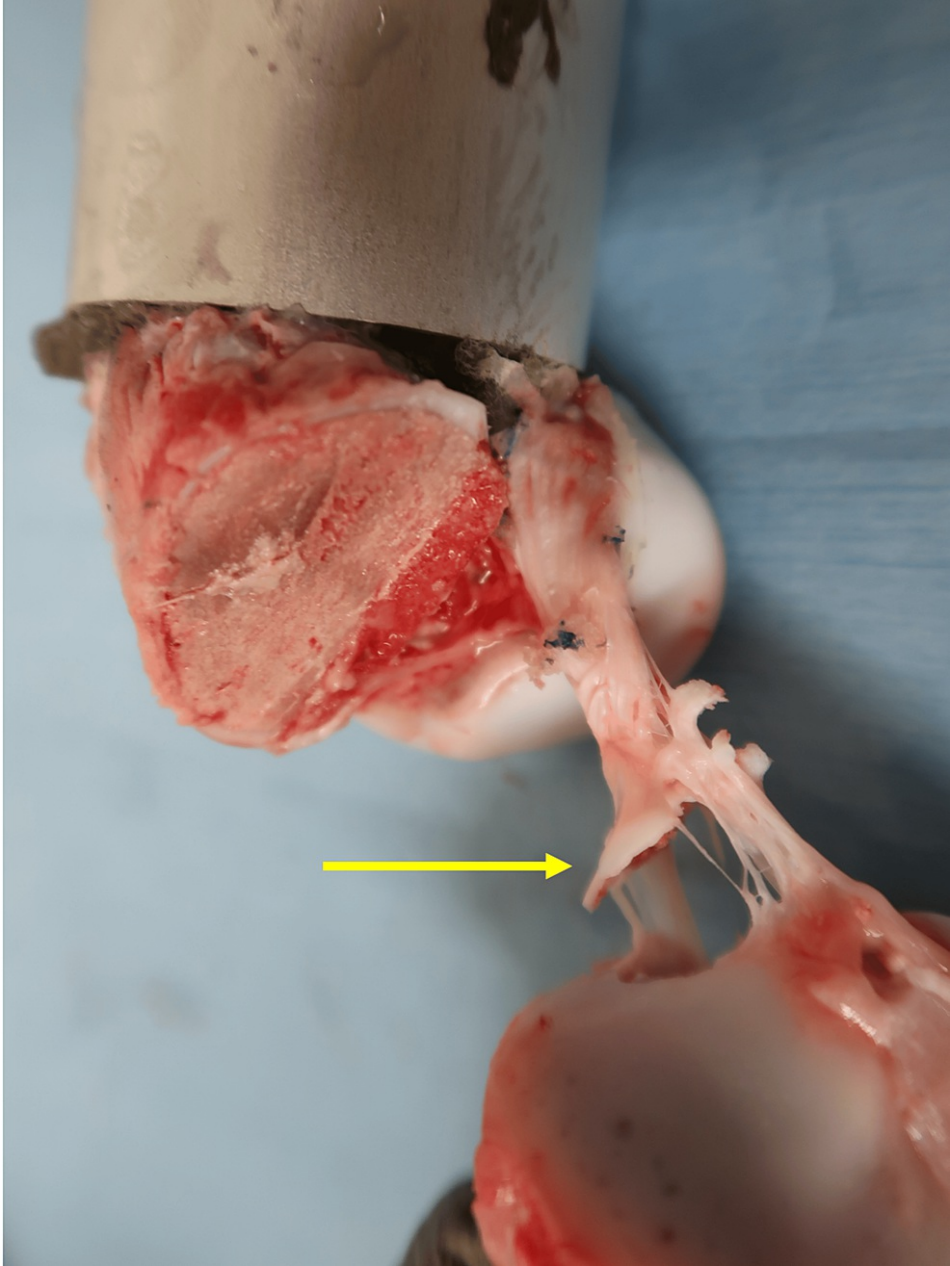
Avulsion at the tibial side of the anterior cruciate ligament attachment after tensile testing Yellow arrow indicates avulsion at the tibial side of the anterior cruciate ligament attachment.

**Figure 6:**
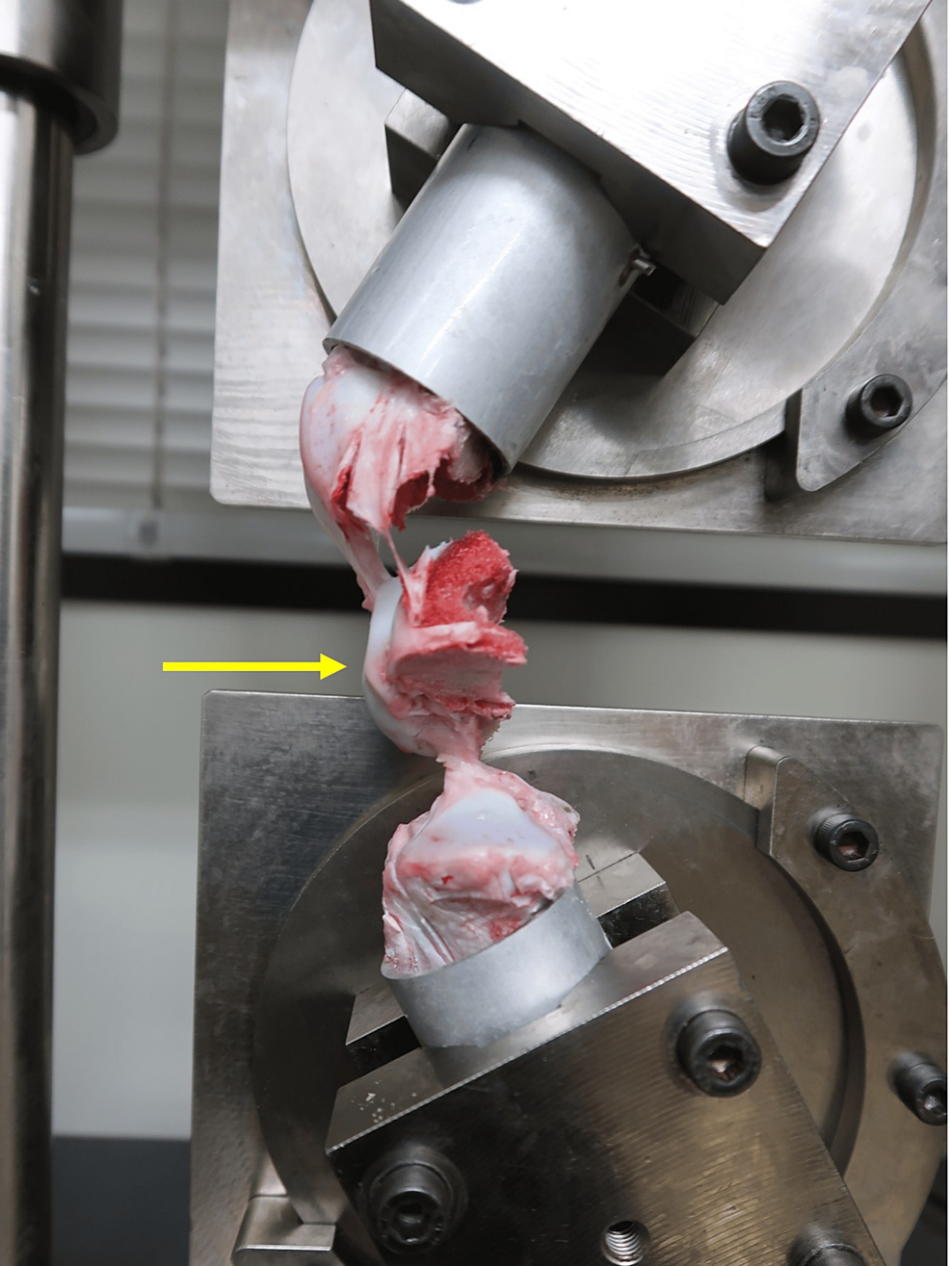
Distal femoral condyle fracture after tensile testing Yellow arrow indicates distal femoral condyle fracture.

Regarding the failure modes in tensile testing, ACL avulsion from the femoral attachment was observed in all FAT composites in the Vulcan group. Conversely, one case of ACL avulsion from the tibial attachment and one case of distal femoral condyle fracture were observed in the Werewolf group (P = 0.46) (Table 2 and Figures 4-6).

**Table 2:** The failure mode at the time of tensile testing and the size of avulsion UF, uncalcified fibrocartilage; CF, calcified fibrocartilage; LB, Lamellar bone; SB, subchondral bone.

	The layer of the avulsion				
	Intact	UF	CF	LB	SB
Vulcan (n=7)	0	1	1	1	4
Werewolf (n=7)	1	1	1	3	1

## Discussion

Despite changes in length during cyclic loading, this study clarified that the upper yield load of the ACL following partial ACL femoral attachment debridement varied depending on the ablation devices used, with no variation in the maximum load.

In shoulder surgery, thermal denaturation of collagen in the ligamentous tissue may enhance arthroscopic shoulder stabilization. A previous study also revealed that RF electrothermal contraction decreases laxity but significantly alters viscoelastic properties, posing a risk of recurrent stretch-out under “physiological” loading [22]. Another study used an electrothermal device; however, our results were not comparable to the previous findings in terms of comparable change in length during cyclic loading and mechanical properties, except for the upper yield load. One of the plausible reasons is that we ensured that the probe applied perpendicular to the fan-like extension fibers would not damage the mid-substance of the ACL throughout the procedure. This was ensured because RF application in knee surgery weakens the biomechanical properties of the native and elongated cruciate ligaments [23]. In addition, Wang et al. stated that the ablation mode may be the most suitable mode for knee cartilage dissection as it induces less thermal radiation damage than the coagulation mode; therefore, we selected the ablation mode [24]. Accordingly, the results may vary if coagulation is performed. Kondo et al. compared the acute effects of electrothermal shrinkage on the biomechanical properties of the ACL and concluded that RF energy application to the specimens caused both shortening and weakening according to the magnitude and duration of the application [16]. The magnitude of RF energy might affect the damage around fan-like extension fibers and surrounding tissues.

A few studies have examined the penetration depth of RF devices. Khoury et al. used bipolar RF on the articular cartilage of bovine-matched knees and evaluated the effect of ablation power outputs on the penetration depth. They reported that the higher the power settings, the deeper the penetration [25]. Edwards et al. compared the effects of bipolar and monopolar RF modes on human osteochondral sections and reported that bipolar devices lead to significantly higher rates of chondrocyte death than monopolar devices [26]. Wang et al. compared the effects of coagulation and ablation in the bipolar RF mode under low and high settings and reported that compared with high-power ablation settings, both coagulation and low-power ablation settings resulted in deeper thermal injury [20]. Amiel et al. examined chondrocyte viability following the application of a bipolar RF probe on fresh bovine articular cartilage. They revealed that the probe enabled a well-controlled debridement with smooth edges and a defined margin of chondrocyte death extending to a depth of 100-200 μm [27]. However, no studies have compared electrothermal devices with electrolyte plasmalization devices to date; thus, we examined the mechanical properties of the ACL after generating ablation from two different devices.

A femoral bone tunnel was created at the proximal end of the remaining ACL remnant tissue during RTP-ACL reconstruction [2, 28]. Liu et al. described that decreased bone mineral density was observed in the affected knee following ACL reconstruction compared to the healthy knee [29]. The quality of the cancellous bone must be maintained so as not to interfere with the tendon-bone integration and to prevent bone tunnel enlargement. Therefore, in future studies, we will evaluate whether the devices used for partial femoral ACL stump debridement in RTP-ACL reconstruction affect the clinical outcomes, graft maturation, and degree of femoral tunnel enlargement after surgery.

Limitations

This study has several limitations. First, a porcine model was used. The differences in cancellous bone properties between young human knees and porcine knees may have influenced the mechanical properties of the ACL after partial debridement. However, porcine knees were reported to be similar to human knees in many aspects [6]. Second, we could not determine the influence on biological healing responses due to ex vivo nature of this study. Third, open surgeries, not arthroscopic surgeries, were performed. Fourth, a limited number of specimens and implants were available for use, which decreased the available sample size in each group.

## Conclusions

As for clinical relevance, when performing RTP-ACL reconstruction, surgeons must consider that the device used for partial femoral ACL stump debridement may affect the mechanical properties of the ACL remnant tissue. The mechanical properties of the ACL following partial ACL femoral attachment debridement for RTP-ACL reconstruction were better when an electrolyte plasmalization device was used.
